# Natural Biocombinatorics in the Polyketide Synthase Genes of the Actinobacterium Streptomyces avermitilis

**DOI:** 10.1371/journal.pcbi.0020132

**Published:** 2006-10-06

**Authors:** Holger Jenke-Kodama, Thomas Börner, Elke Dittmann

**Affiliations:** 1 Department of Molecular Ecology, Institute of Biology, Humboldt University, Berlin, Germany; 2 Department of Genetics, Institute of Biology, Humboldt University, Berlin, Germany; University of Cambridge, United Kingdom

## Abstract

Modular polyketide synthases (PKSs) of bacteria provide an enormous reservoir of natural chemical diversity. Studying natural biocombinatorics may aid in the development of concepts for experimental design of genes for the biosynthesis of new bioactive compounds. Here we address the question of how the modularity of biosynthetic enzymes and the prevalence of multiple gene clusters in *Streptomyces* drive the evolution of metabolic diversity. The phylogeny of ketosynthase (KS) domains of *Streptomyces* PKSs revealed that the majority of modules involved in the biosynthesis of a single compound evolved by duplication of a single ancestor module. Using Streptomyces avermitilis as a model organism, we have reconstructed the evolutionary relationships of different domain types. This analysis suggests that 65% of the modules were altered by recombinational replacements that occurred within and between biosynthetic gene clusters. The natural reprogramming of the biosynthetic pathways was unambiguously confined to domains that account for the structural diversity of the polyketide products and never observed for the KS domains. We provide examples for natural acyltransferase (AT), ketoreductase (KR), and dehydratase (DH)–KR domain replacements. Potential sites of homologous recombination could be identified in interdomain regions and within domains. Our results indicate that homologous recombination facilitated by the modularity of PKS architecture is the most important mechanism underlying polyketide diversity in bacteria.

## Synopsis

Modular polyketide synthases (PKSs) of bacteria are multifunctional enzymes providing a molecular construction plan for the stepwise generation of polyketides of high structural complexity. Natural products of the polyketide class belong to the most important medicines used for the treatment of infectious diseases and cancer. The genetic “programming” of the enzymes determines the choice of different carbon units, the reduction state, and the stereochemistry of the polyketide chain. The modular architecture of PKS enzyme systems lends itself to rational engineering in the laboratory using so-called biocombinatorics approaches. *Streptomycetes* are soil bacteria typically comprising multiple PKS gene clusters. Jenke-Kodama, Börner, and Dittmann have addressed the question whether this prevalence of repetitive PKS modules within a single genome has an impact on the diversification of the polyketide products. Using phylogenetic approaches, the authors provide evidence that homologous recombination has led to exchange, loss, and gain of domains and domain fragments and hence to a natural “reprogramming” of the PKS assembly lines. These data are not only interesting from the evolutionary point of view but might also help to improve protocols for PKS engineering that are being developed for the synthesis of new bioactive compounds and libraries.

## Introduction

**Figure 1:**
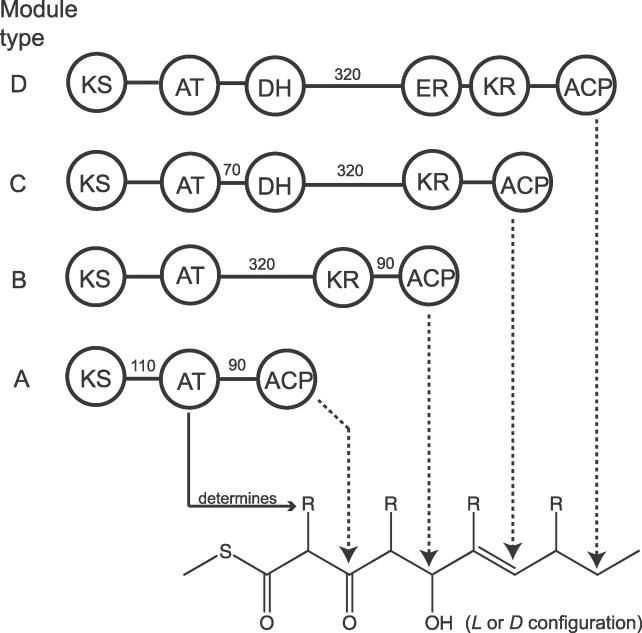
The Different Module Types of Modular PKSs and Their Influence on the Structure of the Polyketide Backbone The numbers written between domains give the typical length of the respective interdomain region in terms of amino acid residues. ER, enoylreductase.

Secondary metabolism shows an extraordinary variety of chemical structures. One major class of natural products are the polyketides, which include a wide range of pharmaceutically important compounds with antibacterial (e.g., erythromycin), immunosuppressive (e.g., rapamycin), and anticancer (e.g., epothilone) activities [1]. Polyketides are produced by different types of synthases [1]. Modular type I polyketide synthases (PKSs) of bacteria are multifunctional enzymes providing an impressive construction plan for the assembly of complex structures from simple carbon building blocks. The chemical steps of chain extension and correspondingly the enzymatic activities are strikingly similar to those of fatty acid synthases [2]. The active sites of type I PKSs are organized linearly into modules, such that each module catalyzes one cycle of elongation. A minimal module contains a ketosynthase (KS), an acyltransferase (AT), and an acyl carrier protein (ACP) domain. The specificity of AT for malonyl-CoA, methylmalonyl-CoA, or other α-alkylmalonyl-CoAs determines which carbon extender is used. Since the latter two substrate types have a chiral center, their incorporation gives different stereoisomers of the prolonged polyketide chain. After condensation, the oxidation state of the β-carbon is either kept as a keto group or modified to a hydroxyl, methine, or methylene group by the optional activity of ketoreductase (KR), dehydratase (DH), and enoylreductase (ER) domains (Figure 1). Further variability comes from the existence of two types of KR domains that create different stereoisomers regarding the chiral β-carbon [3]. Although there are only four different module architectures, which are classified here as type A, B, C, and D (Figure 1), the possibility of combining the different variants in a permutational manner gives an enormous diversity of polyketide structures. Theoretically, a PKS system comprising six elongation modules could produce more than 100,000 possible structures [4].

Ever since the modular principle of the PKS biosynthesis machinery was dissected, scientists were attracted by its obvious combinatorial potential. Different strategies were tested for the generation of “unnatural” product libraries. Novel polyketides were generated by adding, deleting, or exchanging domains within modules, or new products were obtained by recombination of entire modules from different pathways and host strains [1]. These biotechnological approaches can be taken as an attempt to reproduce the events that have shaped PKS clusters during evolution. It has been suggested that the evolution of the multimodular structure of PKSs can be attributed to repeated rounds of gene duplication, resulting in the addition of modules either as gene fusions or in the form of new separate proteins integrated into the assembly line [5]. The diversity of differently programmed PKSs could have been achieved by subsequent exchange of modules. However, it has not been shown yet which kinds of replacements really happen in naturally occurring systems, particularly which components, modules, single domains, or fixed domain groups are actually exchanged to build up new assembly lines thereby creating differently programmed PKSs.

The purpose of this study was to obtain insights into the evolution of metabolic diversity by investigating to what extent the modular architecture of PKS genes allows for natural biocombinatorics. A better understanding of how bacteria benefit from the modularity of multi-enzyme systems may also provide new lessons for experimental biocombinatorial approaches. As the model organism we used the actinobacterium *Streptomyces avermitilis,* taking advantage of three factors that allow for an extensive analysis. First, the complete sequence of the genome of S. avermitilis has been determined [6]. Second, this genome encodes the largest number of PKSs of all bacterial genomes that are currently available in databases, and third, the majority of modules can be assigned to the biosynthesis of three characterized polyketide compounds, avermectin (*ave*), oligomycin (*olm*), and a polyene macrolide (*pte*) [6].

## Results/Discussion

### PKS Clusters of S. avermitilis and Their Phylogenetic Position in the *Streptomyces* Context

**Figure 2:**
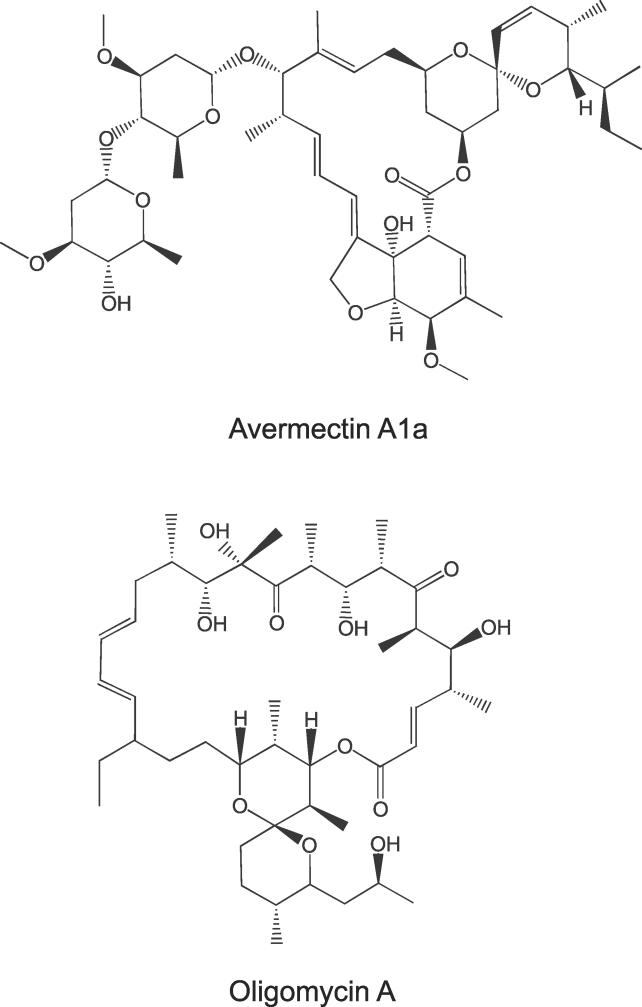
Representative Structures of Secondary Metabolites Classes Produced by the Large PKSs of S. avermitilis The avermectin and oligomycin structures are examples of the respective compound groups. The exact structure of the polyene macrolide compound is not known.

The genome of S. avermitilis contains eight type I PKS gene clusters [6]. The clusters involved in avermectin, oligomycin*,* and polyene macrolide biosynthesis each span between 80 kb and 100 kb and represent 86% of the 51 PKS modules encoded by the strain. The structures of *avermectin* and *oligomycin* are shown in Figure 2. The remaining clusters are much smaller with a length of only 8 kb to 17.5 kb. Within this group, only the two *pks5* modules show high amino acid sequence similarity with the three large clusters and were included in the further analyses.

**Figure 3:**
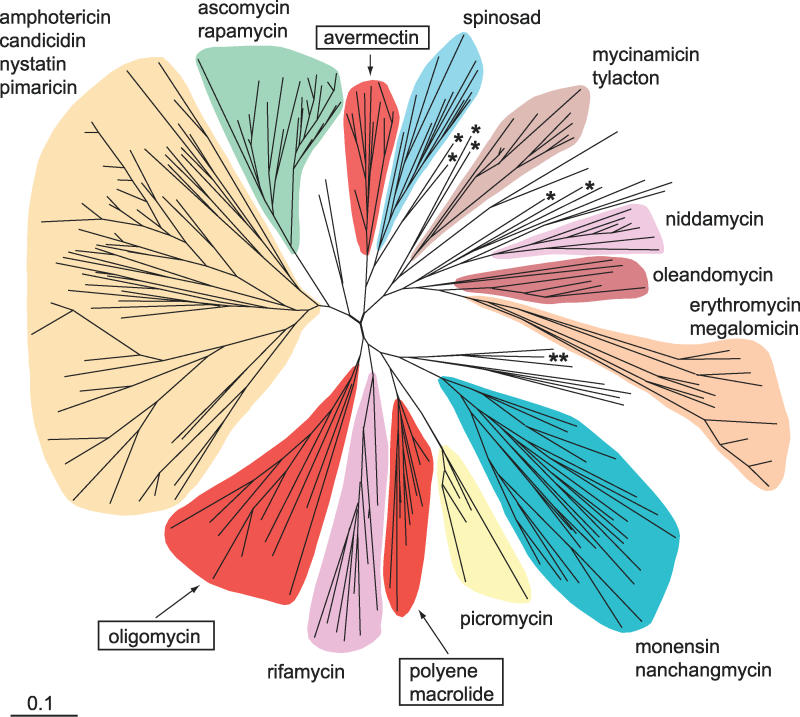
Phylogeny of the KS Domains of Selected PKS Clusters from *Streptomyces* Strains The tree was inferred by Bayesian estimation using amino acid sequences. The domains belonging to the three large PKS clusters of S. avermitilis are highlighted in red and marked by arrows. KS domains that are located outside the main oligomycin and polyene macrolide clades are labeled with a single asterisk and double asterisks, respectively.

To assess the evolutionary context of the S. avermitilis PKS domains, we integrated their KS domains into a dataset of KS domains from 17 characterized PKS pathways of *Streptomyces* species and subjected these data to phylogenetic analysis. The tree reconstruction (Figure 3) shows that the majority of domains are grouped in cluster-specific clades under a reliable node. The detailed tree with sequence names and clade probability values is in Figure S1. The KS domains of the *ave* and the *pte* cluster each form a homogenous group with only one exception for the latter cluster, indicating that the vast majority of KS domains are the outcome of repeated gene duplications. In addition, gene conversion events within a given cluster may have contributed to the observed pattern by homogenizing the sequences. A common clustering of KS domains was also seen for the majority of the other *Streptomyces* pathways investigated. Most of the *olm* sequences are likewise located in a separate cluster, but there are six domains that seem to be phylogenetically more closely related to PKS clusters of other streptomycetes. Part of this topology can be explained as the result of horizontal gene transfer, as it was proposed for the amphotericin, nystatin, and pimaricin synthases based on the striking conformity of the cluster configurations and conspicuous GC content [7]. In general, however, there is no necessity to imply horizontal gene transfer events to explain imperfect clustering patterns, which appear as mixed clusters or relatively separated branches, such as in the case of the *olm* KS domains. Instead, the possibility should be taken into account that the PKS multigene family existed before the speciation processes, resulting in the recent diversity of the *Streptomyces* species. The imperfect clustering pattern may arise from “birth-and-death evolution,” which was detected in a considerable number of multigene families [8]. This model assumes that genes are created by gene duplications and that only some of them are maintained for a long time, whereas others are inactivated and deleted eventually. The involvement of “birth-and-death evolution” is supported by the existence of PKS-like genes in the S. avermitilis genome that are probably nonfunctional due to deleterious mutations and appear to be fragmented remnants of once functional clusters (unpublished data).

Taken together, the phylogenetic analysis of KS domains from streptomycetes indicates that individual pathways have predominantly evolved by duplication of single ancestor modules. We have observed similar relationships of KS domains for selected pathways of myxobacteria and cyanobacteria in a previous phylogenomic study [9]. We may therefore conclude that duplication is a common evolutionary scenario that has led to modularization of biosynthetic pathways and that the evolutionary principle assessed in this study is not limited to *Streptomycetes*.

### Phylogenetic Analysis of Domains and Global Replacement Patterns

**Figure 4:**
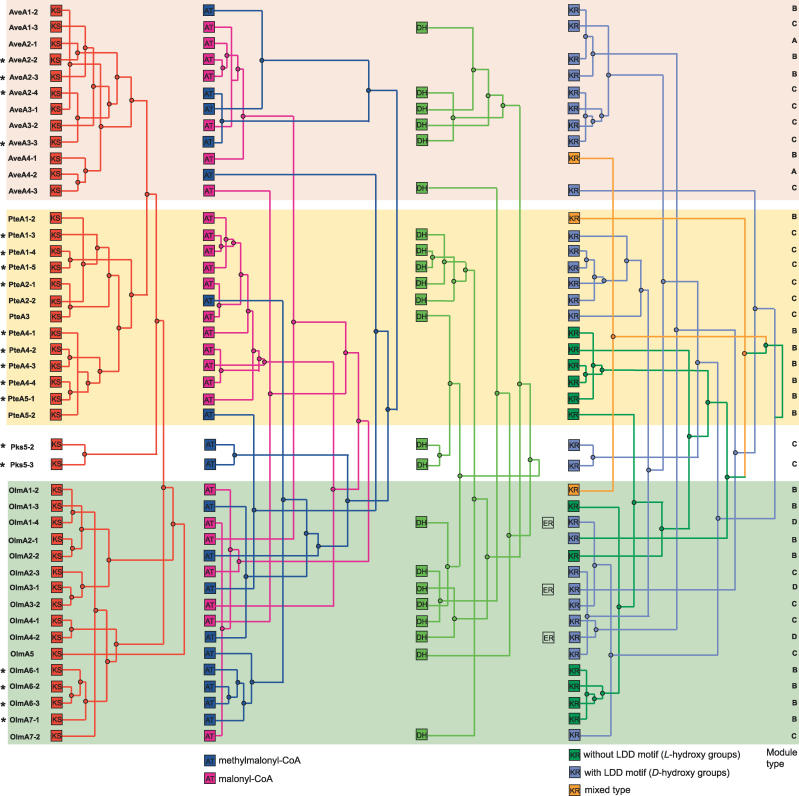
Phylogenies of the Different PKS Domain Types from S. avermitilis Projected onto the Cluster Structure Modules that show complete congruity in all their domains are marked by asterisks on the left. The different subtypes of AT as well as KR domains are represented by different colors. The module types specified on the right are as in Figure 1.

We performed a phylogenetic reconstruction of the different domain types in the three large clusters and the *pks5* genes. Figure 4 shows an integrated scheme, which projects the trees of KS, AT, DH, and KR domains as reconstructed by Bayesian inference (BI) on to the module structure. The parsimony analysis resulted in very similar tree topologies and can be found for comparison together with the Bayesian trees in Figure S2. It was not possible to obtain a reliable phylogeny of ACP domains due to their shortness and high similarity to each other. For all subsequent analyses we used only sequences of clades that were reproducible by both methods to avoid potential problems of a single reconstruction method. The trees in Figure 4 display characteristic relationships depending on the domain type. The tree of AT domains consists of two main clades, the malonyl-CoA–using domains and those using methylmalonyl-CoA. This substrate-specific clustering is always found for AT domains and reflects the early evolutionary separation of the two domain types [9]. The tree of KR domains is also built up from two main groups, which correspond to the functionally distinguishable KR subtypes that were originally found by sequence comparisons [3].

We could classify 15 modules as being nonmosaic (marked by asterisks in Figure 4), i.e., they show complete congruence in all their domains with at least one other module. These modules can be interpreted as the direct result of gene duplications after which no further changes have happened. On the other hand, 65% of the modules show phylogenetic incongruities. Interestingly, the nonfitting “foreign” stretches are not equally distributed over the domain types. As seen in the overall tree of *Streptomyces* KS domains (Figure 3), we found that virtually all KS domains of the same cluster can be interpreted as one single clade that was formed from a common ancestor without any mixing between clusters. In contrast to KS, some of the AT domains of one cluster have near common ancestors with ATs of other clusters. The same phenomenon can be seen for DH and KR domains, albeit a cluster-specific ancestor connects the majority of domains. In conclusion, the global exchange patterns indicate recombination events between different PKS clusters encoded by a single strain. Strikingly, the evidence for sequence replacement is confined to domain types that exist in enzymatically different variants and whose absence or presence leads to a change in the chemical structure of the product. In the following sections we dissect examples of recombination for the different domain types.

### Replacement of AT Domains

**Figure 5:**
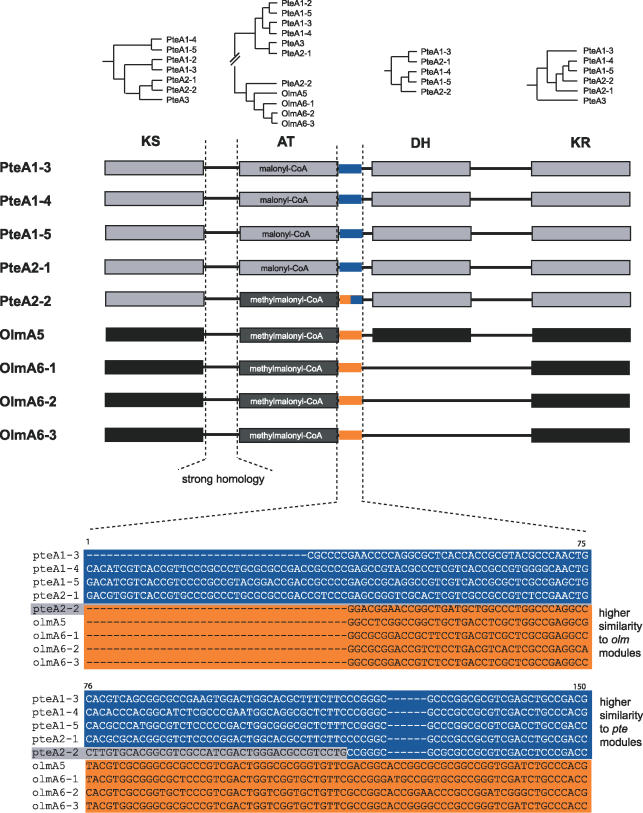
Replacement of an AT Domain The incongruent phylogenetic clustering of the PteA2–2 AT domain is displayed in the miniaturized trees. AT–DH interdomain regions are highlighted in blue and yellow to show the hybrid character of the PteA2–2 interdomain region.

Changing the type of the carboxylic acid monomer to be incorporated into the polyketide chain is an important means to create product versatility. The successive modules PteA1–3, PteA1–4, PteA1–5, PteA2–1, and PteA2–2 all are of type C, thus having the optional DH and KR domains. KS, DH, and KR domains of each module belong to the same phylogenetic group, whereas there is an incongruity regarding the AT domains. PteA2–2 is the only module in the set using methylmalonyl-CoA, all the other ones show substrate specificity for malonyl-CoA (Figure 5). As malonyl-CoA–activating and methylmalonyl-CoA–activating domains were separated early in evolution and form distinct clades in phylogenetic trees (see [9] and Figure S2C and S2D), an alteration of the substrate specificity by point mutations is very unlikely. Rather, the phylogenetic incongruity can be explained by a recombination event.

The closest neighbors of the PteA2–2 AT are those of OlmA5 and the OlmA6 modules. The interdomain regions upstream of the AT domains show high similarity over their whole length. Downstream of AT there is an area of high sequence similarity. Remarkably, the AT–DH interdomain region of PteA2–2 is a hybrid sequence: in the 5′ part it is more similar to the *olm* sequences, whereas farther downstream a higher similarity to *pte* sequences was observed. This argues for recombination breakpoints being located in the interdomain regions in front of and behind the AT domains. A very similar constellation was found within the *ave* cluster regarding the modules AveA1–3 and AveA3–2 showing specificity for malonyl-CoA, AveA2–4, and AveA3–3 using methylmalonyl-CoA.

### Changing the Reduction Level of the Polyketide Chain

**Figure 6:**
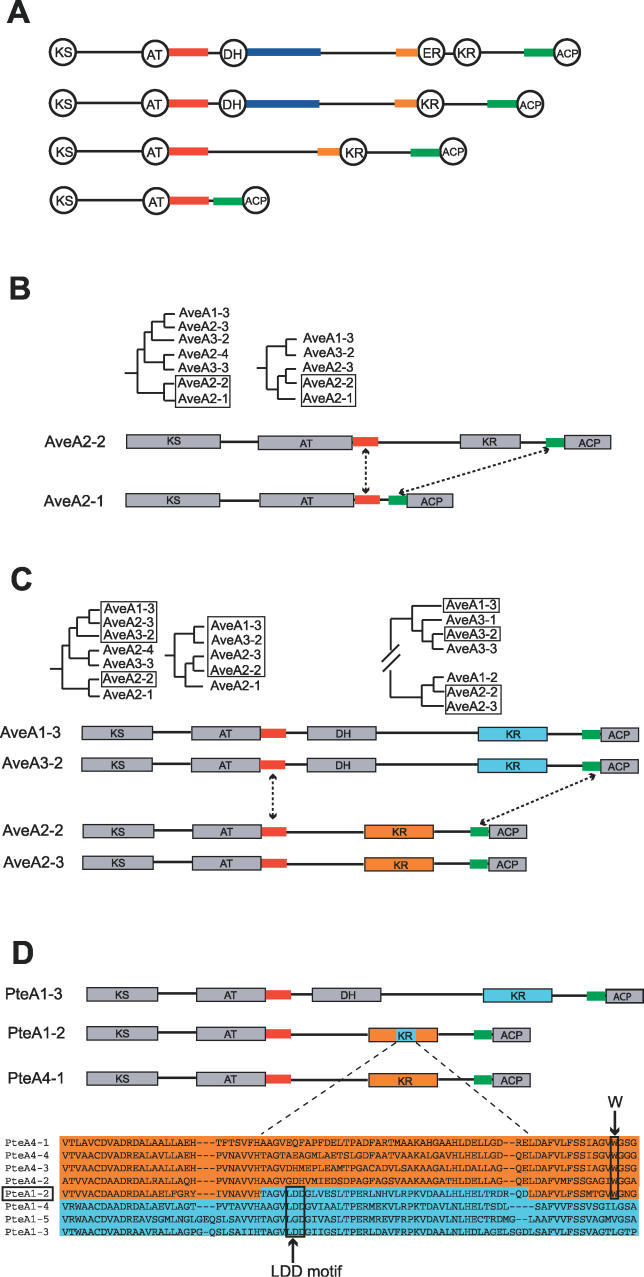
Reduction Level Changes by Recombinatorial Sequence Replacements (A) Homologous sequence stretches in the interdomain linkers of the different module types. (B) Loss or gain of a KR domain. (C) Exchange of a DH–KR domain unit. (D) Creation of a mixed KR domain type by recombination. Partial amino acid sequences are depicted in blue and orange to show the hybrid character of the PteA1–2 KR domain.

The sequence homology patterns found in the different module configurations provide clues for the actual processes that occurred during evolution in the S. avermitilis genome (Figure 6A). Sequence homology is found in all KR–ACP interdomain regions, the 3′ part of which is also part of the AT–ACP interdomain region of the basic module type A. The homologous sequence stretches between AT and DH domains are also found in the 5′ region of the AT–KR and the AT–ACP interdomain regions. Whereas the first 400 bp of the long DH–ER and DH–KR regions show high similarity, this is not the case for AT–KR connecting sequences.

The possibility to interconvert module types A and B is exemplified by analysis of the type A module AveA2–1. The KS and AT are phylogenetically closely related to the respective domains of the module AveA2–2, which belongs to type B, having an additional KR domain (Figure 6B). The comparison of the AT–ACP and AT–KR interdomain regions, respectively, showed a nearly identical sequence segment of about 150 bp, which is exclusively found in modules of the *ave* cluster. A second homologous sequence stretch was found in the posterior part of the KR–ACP interdomain region of AveA2–2 and the AT–ACP interdomain region of AveA2–1. This constellation can thus be interpreted as the result of the loss of a large sequence section, which was situated between AT and ACP and contained a KR domain. Alternatively, a type A module could have been changed to type B by integrating a KR domain together with the characteristically long interdomain region in front of it. For both these scenarios, the recombination breakpoints were probably located in the respective interdomain regions, which surrounded the replaced sequence element.

Recombinational transfer of complete DH–KR units can be deduced from comparing a set of four modules of the *ave* cluster (Figure 6C), two of type B and two of type C. Whereas they show complete congruity with regard to their KS and AT domains, the KR domains belong to different phylogenetic groups. This sequence ensemble likely resulted from an exchange of a DH–KR unit with a single KR domain or vice versa*.* This type of conversion is supported by the distinct homology of AT–DH interdomain regions and the first approximately 200 bp of the region between AT and KR. Similarly, the second module of PteA1 likely has lost the DH domain by recombination with a module of PteA4 (Figure 6D). This suggestion is strongly supported by the fact that the KR domain of PteA1–2 does not belong to the same type as the KR of the other PteA1 modules, which are exclusively of the B type. Instead, it shares very high amino acid similarity with the KR domain type of the PteA4 modules, namely 92% within the first 80 positions and 91% in the last 50 positions. The sequence in between, however, seems to stem from a different KR type as it shows 87% similarity with the other KRs of PteA1. Like these domains, it has the LDD amino acid motif being typical for the D configuration–producing KR domains. Probably the original DH–KR unit was at first replaced by a KR unit concomitantly changing the KR type. A second recombination transformed the domain's center part back into the original type. This exemplifies that the borders of the underlying recombination events are not restricted to interdomain regions, but may be also located in homologous stretches of the domains themselves. We have found hybrid KR domains in each of the three major PKS pathways of S. avermitilis (Figure 4). Interestingly, at least one of these domains, namely the KR of the module AveA4–1, was shown to be nonfunctional in the biosynthesis of avermectin [10]. This exemplifies that recombination events do not always lead to the diversification of modules, but may also lead to the loss of domain functionality.

### Natural versus Laboratory Biocombinatorics

Our analysis demonstrates that the majority of PKS modules in S. avermitilis were formed by recombination processes that affected regions that are responsible for substrate selection and for the reductive reactions that shape the polyketide backbone. Regarding the types of replacement processes, this truly natural biocombinatorics matches diverse efforts aimed at the production of new compounds in the laboratory. Exchange of AT domains [11–14], substitution of an AT–KR–ACP unit against AT–ACP [15], and replacement of a KR domain by an intact DH–KR unit from another module [15] have been reported, although they seem not to be suited for high-throughput production of novel compounds. Every single step may turn out to be laborious and prone to failures caused by nonfunctional new combinations of domains and modules.

A new method has been introduced recently [16] that might approach natural biocombinatorics principles much more than any earlier trial. This method allows for an adaptation of the codon usage to a suitable expression host like Escherichia coli and the introduction of unique restriction sites flanking domains, linkers, and modules. Thus, it is possible to create easily exchangeable building units. So far the experimental evaluation of this new method has been restricted to create new combinations of complete modules. It would be highly interesting to utilize the method to interchange single domains or certain domain units between different modules, because this procedure would correspond to the kinds of domain replacements that we have detected in the PKS genes of S. avermitilis. In this context it may be interesting to note that we found no evidence for a KS domain exchange between individual PKS pathways of S. avermitilis. This could indicate that congeneric KS domains cooperate better than evolutionarily distinct KS domains within an enzyme complex.

The fundamental difference between natural and experimental biocombinatorics is that the bacterium uses recombination, whereas the experimental method is based on restriction and re-ligation.

In principle, it should be possible to design an experimental approach that is based on recombination. Previous studies describing experimental recombination have frequently used undamaged homologous gene copies for the repair of mutated genes (for reviews see [17,18]). But even without the need for repair and the underlying selective pressure, gene conversion events leading to the diversification of surface antigens in pathogenic bacteria have been verified experimentally [18]. The frequency of the proposed recombination events in PKS genes can be estimated to be much lower than antigenic variations on the surfaces of pathogenic bacteria. To verify the impact of PKS recombination in a limited number of generations, therefore, selection pressure is needed. An experimental approach would be most promising when a polyketide product provides a specific advantage to the producing bacterium under certain conditions and allows for an easy selection. The corresponding PKS multi-enzyme could be mutated, leading to the loss of functionality for individual domains. If the strain contains multiple PKSs, it should be able to repair the damaged gene fragments. To increase the frequency of recombination, it would be reasonable to use mutant strains lacking functional mismatch repair genes [19]. This type of experiment could not only show the possibilities and limits of PKS recombination but could also answer the question whether the exchange of gene fragments occurs reciprocally or nonreciprocally.

### Recombination as the Basis of PKS Variability

Uncovering the mechanisms of protein evolution and explaining the wealth of enzymatic and metabolite diversity in general is still a great challenge. The idea that promiscuous activity in a protein can provide a selective advantage, thereby enabling the organism to survive and to further evolve, was formalized 30 years ago [20]. Since then, many examples for such processes have been described. The task to unravel the evolutionary mechanisms underlying the evolution of secondary metabolism is equally challenging because of the vast diversity of natural products. Firn and Jones proposed a simple evolution-based model in order to create a framework that can explain the existence of this chemical diversity and how it is generated, the so-called Screening Hypothesis [21,22]. This model acknowledges the fundamental fact that a biomolecular activity, i.e., the capability to interact with a protein target with high affinity in a specific and noncovalent way, is a very rare property. It should be advantageous for an organism to possess a synthesis system that favors the production of multiple products. Based on these considerations, it has been predicted that enzymes of secondary metabolism typically have broad substrate specificity and are organized in branched and matrix pathways. Both the evolution of broad substrate specificity and of altered substrate specificity operates on the active centers of enzymes and originates from point mutations. A typical example of this kind of process is the evolution of stilbene synthases, which developed several times independently from chalcone synthases [23]. However, reliance on changes in the active centers means that these systems face the same evolutionary restriction as other proteins, namely the limitation of sequence diversity.

Modular PKSs demonstrate that there is a second very efficient way to create extreme versatility. Though the KS component of modular PKSs somehow fulfills the expected broad substrate specificity, the main invention of enzymatic assembly line processes is the possibility of combinatorial plethora by using homologous recombination. The importance of recombination processes for providing product versatility has already been described for other systems. Phylogenetic analysis of the microcystin biosynthesis cluster in cyanobacterial strains of the species *Microcystis* revealed that recombination was involved in their evolution [24]. The important role of homologous recombination for generating antigenic diversity in pathogenic bacteria was already emphasized. All the proteins analyzed in this context are structural components of the outer membrane of pathogens [18]. Modular PKSs are the first example of an enzyme system whose flexibility is apparently governed by extensive recombinational processes, leading to duplication and domain transfer.

We have determined selection within the PKSs of S. avermitilis in terms of the ratio of nonsynonymous substitutions (d_N_) and synonymous substitutions (d_S_) per site. For all domain types, d_N_ was significantly lower than d_S_, indicating purifying selection (unpublished data). Furthermore, we carried out sliding-window analyses for the complete sequence sets of each module type in order to detect regions of potential positive selection. However, no such regions could be identified in any module type (unpublished data). Thus, their developmental potential is not based on point mutations followed by positive selection of a new enzyme function. Rather, there was only purifying selection, which secures the functionality of the components. The bacterium equipped with its recombination machinery is capable of rebuilding existing PKS clusters by many different kinds of rearrangements and additionally by duplications and insertions. In this process, many unfavorable and unproductive changes may happen, but in some phases of cluster evolution positive selective pressure will set in to stabilize and fix a certain configuration within the population due to the usefulness of the respective compound.

This concept also fits well with the observations that strains of *Streptomyces* often produce two chemically different metabolites that act synergistically against a common target [25] and that they possess contingently acting metabolites, i.e., natural products that have similar biological activity, but are independently used by the producers. The existence of two pathways for the production of siderophores in Streptomyces coelicolor is an example of the latter phenomenon [26]. Based on a growing number of examples, it has been proposed recently that such synergy and contingency effects are driving forces in natural product evolution [27]. Oligomycin and the polyene macrolide compound of S. avermitilis both have antifungal activity [27]. The selective pressure acts on whole synthases as a unit and deter the organism from further changes of the cluster structure. The modular architecture together with the efficiency of the underlying (re)combinatorial principle can be interpreted as a very useful evolutionary invention because of its inherent evolvability, allowing for permanent change beyond the limitations of sequence diversity. This explains the seeming paradox why an organism uses such giant synthesis systems encoded by large regions of the genome to produce rather small natural products. In the recently completed genome of the social amoeba *Dictyostelium discoideum,* genes encoding 43 putative PKSs were identified [28]. It would be interesting to analyze the evolution of these eukaryotic PKS clusters and to figure out whether the same sort of evolutionary strategy is followed in this organism.

In the context of cluster evolution by recombination, it will be also interesting to analyze nonribosomal peptide synthetases (NRPSs), the other synthesis system of secondary metabolism being organized in modules, with regard to the impact of recombination on their evolution. NRPSs show intriguing analogies with modular PKSs in their architecture and functional principles, and, moreover, hybrid systems comprising NRPS and PKS components are known [29]. It can be anticipated that the wealth of nonribosomal peptides is also founded in the recombination-based evolutionary plasticity of the underlying biosynthetic machinery.

## Materials and Methods

### Data retrieval.

The amino acid and nucleotide sequences of the PKS clusters of S. avermitilis were retrieved from the S. avermitilis genome website (http://avermitilis.ls.kitasato-u.ac.jp). The sequences of the proteins of the following characterized *Streptomyces* PKS clusters were obtained from public databases via the National Center for Biotechnology Information server (http://www.ncbi.nlm.nih.gov): OleAI, OleAII (oleandomycin, Streptomyces antibioticus), NidA1 – NidA5 (niddamycin, Streptomyces caelestis), MonAI – MonAVIII (monensin, Streptomyces cinnamonensis), TylG1 – TylG5 (tylacton, Streptomyces fradiae), FkbA – FkbC (FK520, Streptomyces hygroscopicus subsp. *ascomyceticus*), RapA – RapC (rapamycin, Streptomyces hygroscopicus), NanA1 – NanA8, NanA11 (nanchangmycin, Streptomyces nanchangensis), PimS0 – PimS4 (pimaricin, Streptomyces natalensis), AmphA – AmphK (amphotericin, Streptomyces nodosus), NysA – NysK (nystatin, Streptomyces noursei), FscA – FscF (candicidin, *Streptomyces* str. FR-008), PikAI – PikAIV (pikromycin, Streptomyces venezuelae), DEBS1 – DEBS3 (erythromycin, Saccharopolyspora erythraea), SpnA – SpnE (spinosad, Saccharopolyspora spinosa), RifA – RifE (rifamycin, Amycolatopsis mediterranei), MycAI – MycAV (mycinamicin, Micromonospora griseorubida), MegAI – MegAIII (megalomicin, Micromonospora megalomicea). Swiss-Prot (http://www.ebi.ac.uk/swissprot) accession numbers of the proteins used in this study are summarized in Table S1.

### Phylogenetic analysis.

All alignments of amino acid sequences were created by using ClustalX [30] and edited manually. Large insertions and deletions were removed. Nucleotide sequences were aligned on the basis of the respective amino acid sequences. The alignment of streptomycetes KS domains comprised 221 sequences showing 358 amino acid positions. Tree reconstructions were performed by using BI and the distance-based neighbor-joining method. The Bayesian estimation was done by means of the MrBayes software version 3 [31] and employed the JTT amino acid replacement model [32]. Site rate heterogeneity was modeled using a gamma distribution with four categories (JTT+γ). Two parallel Metropolis-coupled Markov chain analyses were performed with 10 million generations and four independent chains. The Markov chains were sampled every 100 generations. Clade probability values were calculated from trees retained after a burn-in phase of 6 million generations. Convergence was judged by the run statistics and the average standard deviation of split frequencies between the two parallel Metropolis-coupled Markov chain analyses with a standard deviation limit of 0.05. The neighbor-joining method was conducted using the modules Seqboot, Protdist, Neighbor, and Consens of the PHYLIP software package version 3.65 [33]. Again, the analysis employed the JTT+γ amino acid replacement model. The α-parameter of the gamma distribution was calculated by using the Tree-Puzzle software version 5.2 [34]. Bootstrap analysis was done using 500 pseudo-replicate sequences.

Tree reconstructions for the different PKS domain types from S. avermitilis were conducted by using Bayesian estimation and the maximum parsimony (MP) method. For the Bayesian estimation, a mixed dataset of amino acid sequences and nucleotide sequences was used. The JTT+γ model was applied on the amino acid data and the general time reversible model of nucleotide exchange [35] on the nucleotide data, which had been divided according to codon positions. The analyses were performed in the same way as described above with the following numbers of generations: KS, 4 million generations, burn-in of 2 million generations; AT: 4 million generations, burn-in of 2 million generations; DH, 2 million generations, burn-in of 0.8 million generations; KR: 2.5 million generations, burn-in of 1 million generations. In all cases, the standard deviation limit to judge the convergence state was 0.005. Trees of the burn-in phase were discarded and the consensus trees and clade probability values were calculated from the trees obtained after reaching the convergence state.

The MP analysis of nucleotide sequences was performed using the heuristic search option of the PAUP software version 4.0b [36], with gaps being treated as missing data. Constant and uninformative data were excluded. Branch swapping was done by using the tree-bisection–reconnection option. The final trees were calculated as strict consensus trees of all best trees.

## Supporting Information

Figure S1 Phylogeny of the KS Domains of Selected PKS Clusters from *Streptomyces S*trains as Obtained by BI Clade probability values are given for the main nodes.
(921 KB EPS)

Figure S2 Phylogenetic Trees of PKS Domains from S. avermitilis as Obtained by Using BI and MP Analysis (A) BI tree of KS. (B) MP tree of KS. (C) BI tree of AT. (D) MP tree of AT. (E) BI tree of DH. (F) MP tree of DH. (G) BI tree of KR. (H) MP tree of KR. For the BI trees, clade probability values are given. MP trees were calculated as strict consensus trees of best trees.
(1.7 MB PDF)

Table S1 Swiss-Prot Accession Numbers of the Proteins Used in This Study (59 KB DOC)

## Abbreviations

ACP: acyl carrier protein
AT: acyltransferase
BI: Bayesian inference
DH: dehydratase
KR: ketoreductase
KS: ketosynthase
MP: maximum parsimony
NRPSs: nonribosomal peptide synthetases
PKSs: polyketide synthases
