# Supplementary material for: Natural Biocombinatorics in the Polyketide Synthase Genes of the Actinobacterium Streptomyces avermitilis
Source: PLoS Comput Biol. 2006 Oct 6;2(10):e132. doi: 10.1371/journal.pcbi.0020132 (PMC1592344; doi:10.1371/journal.pcbi.0020132)
Supplement: Supplementary file 1 [file pcbi.0020132.sg002.pdf]

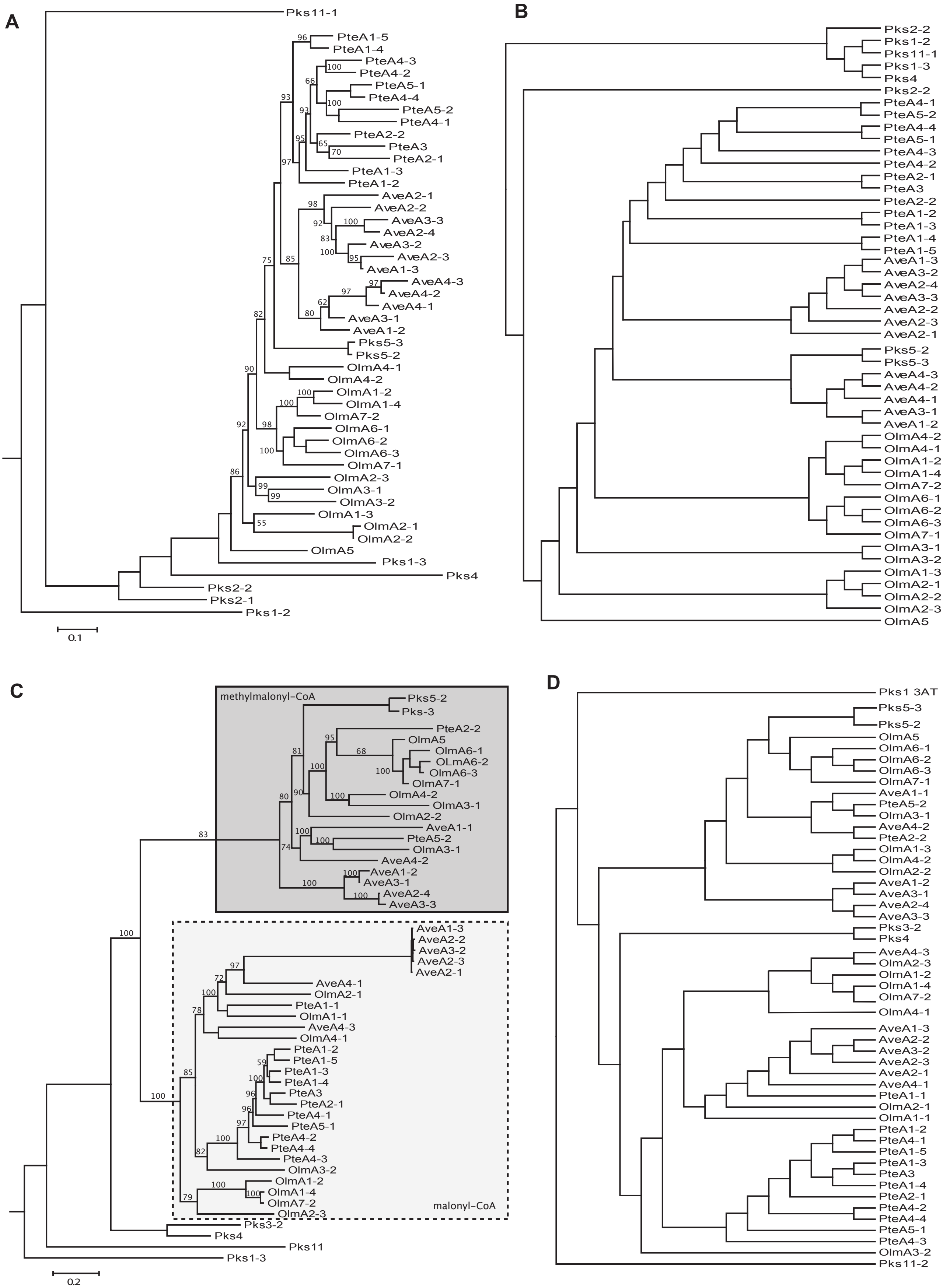

A
Pks11-1
PteA1-5
PteA1-4
PteA4-3
PteA4-2
PteA5-1
PteA4-4
PteA5-2
PteA4-1
PteA2-2
PteA3
PteA2-1
PteA1-3
PteA1-2
AveA2-1
AveA2-2
AveA3-3
AveA2-4
AveA3-2
AveA2-3
AveA1-3
AveA4-3
AveA4-2
AveA4-1
AveA3-1
AveA1-2
Pks5-3
Pks5-2
OlmA4-1
OlmA4-2
OlmA1-2
OlmA1-4
OlmA7-2
OlmA6-1
OlmA6-2
OlmA6-3
OlmA7-1
OlmA2-3
OlmA3-1
OlmA3-2
OlmA1-3
OlmA2-1
OlmA2-2
OlmA5
Pks1-3
Pks4
Pks2-2
Pks2-1
Pks1-2
0.1
B
Pks2-2
Pks1-2
Pks11-1
Pks1-3
Pks4
Pks2-2
PteA4-1
PteA5-2
PteA4-4
PteA5-1
PteA4-3
PteA4-2
PteA2-1
PteA3
PteA2-2
PteA1-2
PteA1-3
PteA1-4
PteA1-5
AveA1-3
AveA3-2
AveA2-4
AveA3-3
AveA2-2
AveA2-3
AveA2-1
Pks5-2
Pks5-3
AveA4-3
AveA4-2
AveA4-1
AveA3-1
AveA1-2
OlmA4-2
OlmA4-1
OlmA1-2
OlmA1-4
OlmA7-2
OlmA6-1
OlmA6-2
OlmA6-3
OlmA7-1
OlmA3-1
OlmA3-2
OlmA1-3
OlmA2-1
OlmA2-2
OlmA2-3
OlmA5
C
methylmalonyl-CoA
Pks5-2
Pks-3
PteA2-2
OlmA5
OlmA6-1
OLmA6-2
OlmA6-3
OlmA7-1
OlmA4-2
OlmA3-1
OlmA2-2
AveA1-1
PteA5-2
OlmA3-1
AveA4-2
AveA1-2
AveA3-1
AveA2-4
AveA3-3
AveA1-3
AveA2-2
AveA3-2
AveA2-3
AveA2-1
AveA4-1
OlmA2-1
PteA1-1
OlmA1-1
AveA4-3
OlmA4-1
PteA1-2
PteA1-5
PteA1-3
PteA1-4
PteA3
PteA2-1
PteA4-1
PteA5-1
PteA4-2
PteA4-4
PteA4-3
OlmA3-2
OlmA1-2
OlmA1-4
OlmA7-2
OlmA2-3
malonyl-CoA
Pks3-2
Pks4
Pks11
Pks1-3
0.2
D
Pks1 3AT
Pks5-3
Pks5-2
OlmA5
OlmA6-1
OlmA6-2
OlmA6-3
OlmA7-1
AveA1-1
PteA5-2
OlmA3-1
AveA4-2
PteA2-2
OlmA1-3
OlmA4-2
OlmA2-2
AveA1-2
AveA3-1
AveA2-4
AveA3-3
Pks3-2
Pks4
AveA4-3
OlmA2-3
OlmA1-2
OlmA1-4
OlmA7-2
OlmA4-1
AveA1-3
AveA2-2
AveA3-2
AveA2-3
AveA2-1
AveA4-1
PteA1-1
OlmA2-1
OlmA1-1
PteA1-2
PteA4-1
PteA1-5
PteA1-3
PteA3
PteA1-4
PteA2-1
PteA4-2
PteA4-4
PteA5-1
PteA4-3
OlmA3-2
Pks11-2

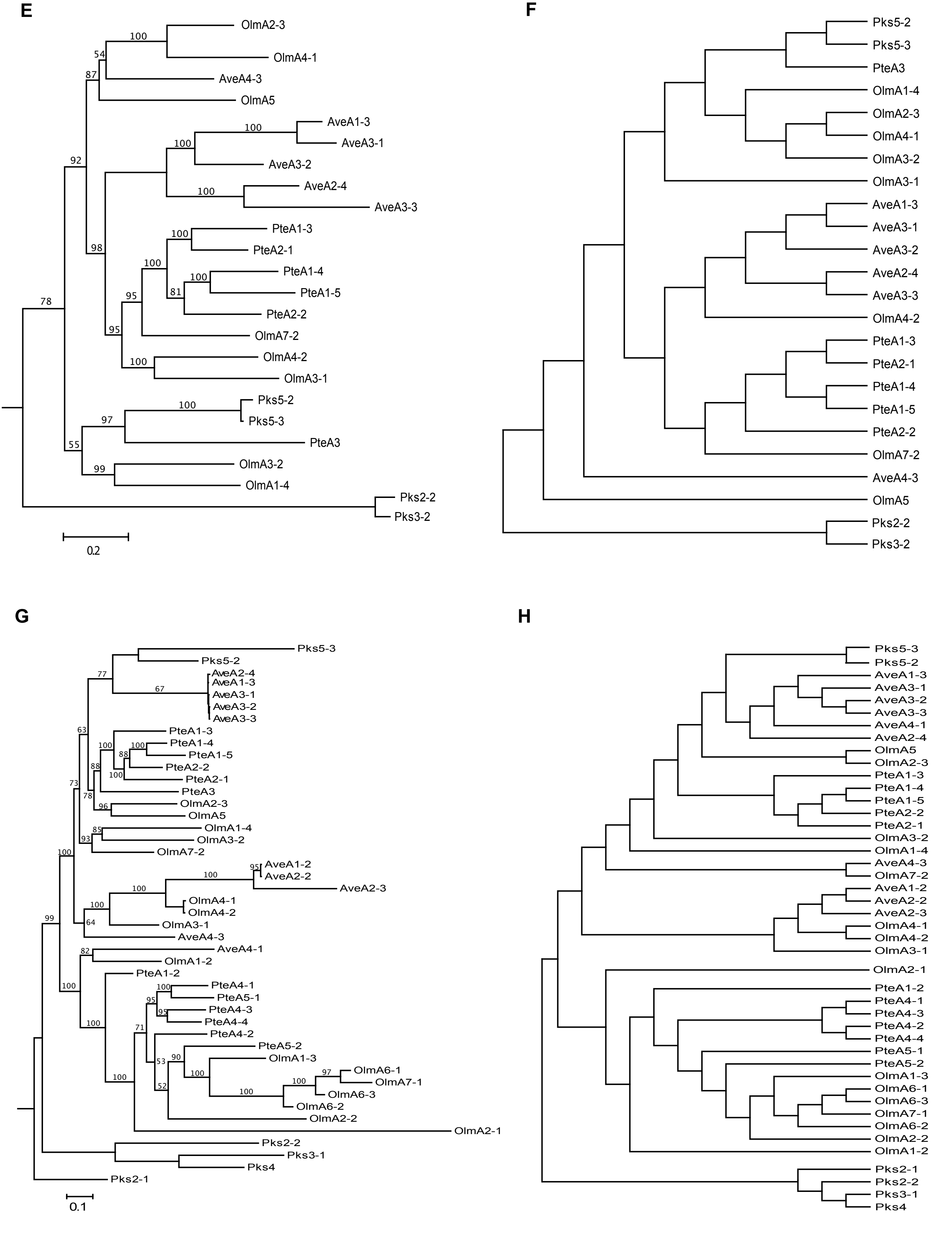
E
OlmA2-3
OlmA4-1
AveA4-3
OlmA5
AveA1-3
AveA3-1
AveA3-2
AveA2-4
AveA3-3
PteA1-3
PteA2-1
PteA1-4
PteA1-5
PteA2-2
OlmA7-2
OlmA4-2
OlmA3-1
Pks5-2
Pks5-3
PteA3
OlmA3-2
OlmA1-4
Pks2-2
Pks3-2
0.2
F
Pks5-2
Pks5-3
PteA3
OlmA1-4
OlmA2-3
OlmA4-1
OlmA3-2
OlmA3-1
AveA1-3
AveA3-1
AveA3-2
AveA2-4
AveA3-3
OlmA4-2
PteA1-3
PteA2-1
PteA1-4
PteA1-5
PteA2-2
OlmA7-2
AveA4-3
OlmA5
Pks2-2
Pks3-2
G
Pks5-3
Pks5-2
AveA2-4
AveA1-3
AveA3-1
AveA3-2
AveA3-3
PteA1-3
PteA1-4
PteA1-5
PteA2-2
PteA2-1
PteA3
OlmA2-3
OlmA5
OlmA1-4
OlmA3-2
OlmA7-2
AveA1-2
AveA2-2
AveA2-3
OlmA4-1
OlmA4-2
OlmA3-1
AveA4-3
AveA4-1
OlmA1-2
PteA1-2
PteA4-1
PteA5-1
PteA4-3
PteA4-4
PteA4-2
PteA5-2
OlmA1-3
OlmA6-1
OlmA7-1
OlmA6-3
OlmA6-2
OlmA2-2
OlmA2-1
Pks2-2
Pks3-1
Pks4
Pks2-1
0.1
H
Pks5-3
Pks5-2
AveA1-3
AveA3-1
AveA3-2
AveA3-3
AveA4-1
AveA2-4
OlmA5
OlmA2-3
PteA1-3
PteA1-4
PteA1-5
PteA2-2
PteA2-1
OlmA3-2
OlmA1-4
AveA4-3
OlmA7-2
AveA1-2
AveA2-2
AveA2-3
OlmA4-1
OlmA4-2
OlmA3-1
OlmA2-1
PteA1-2
PteA4-1
PteA4-3
PteA4-2
PteA4-4
PteA5-1
PteA5-2
OlmA1-3
OlmA6-1
OlmA6-3
OlmA7-1
OlmA6-2
OlmA2-2
OlmA1-2
Pks2-1
Pks2-2
Pks3-1
Pks4
