# Supplementary material for: Natural Biocombinatorics in the Polyketide Synthase Genes of the Actinobacterium Streptomyces avermitilis
Source: PLoS Comput Biol. 2006 Oct 6;2(10):e132. doi: 10.1371/journal.pcbi.0020132 (PMC1592344; doi:10.1371/journal.pcbi.0020132)
Supplement: Supplementary file 2 [file pcbi.0020132.st001.doc]

# Table 1

**Swiss-Prot accession numbers of the proteins used in this study**

| Organism | Polyketide product | Protein | Accession number |
| --- | --- | --- | --- |
| Streptomyces avermitilis | avermectin | AveA1 | Q9S0R8 |
|  |  | AveA2 | Q9S0R7 |
|  |  | AveA3 | Q9S0R4 |
|  |  | AveA4 | Q9S0R3 |
|  | polyene macrolide | PteA1 | Q93H87 |
|  |  | PteA2 | Q93H86 |
|  |  | PteA3 | Q93H85 |
|  |  | PteA4 | Q93H84 |
|  |  | PteA5 | Q93H83 |
|  | oligomycin | OlmA1 | Q93HJ5 |
|  |  | OlmA2 | Q93HJ4 |
|  |  | OlmA3 | Q93HJ3 |
|  |  | OlmA4 | Q93HI8 |
|  |  | OlmA5 | Q93HI9 |
|  |  | OlmA6 | Q93HJ2 |
|  |  | OlmA7 | Q93HJ1 |
| *Streptomyces antibioticus* | oleandomycin | OleAI | Q9KIV4 |
|  |  | OleAII | Q9KIV3 |
| *Streptomyces caelestis* | niddamycin | NidA1 | O30764 |
|  |  | NidA2 | O30765 |
|  |  | NidA3 | O30766 |
|  |  | NidA4 | O30767 |
|  |  | NidA5 | O30768 |
| *Streptomyces cinnamonensis* | monensin | MonAI | Q846X6 |
|  |  | MonAII | Q846X5 |
|  |  | MonAIII | Q846X4 |
|  |  | MonAIV | Q846X3 |
|  |  | MonAV | Q846X2 |
|  |  | MonAVI | Q864X1 |
|  |  | MonAVII | Q846W5 |
|  |  | MonAVIII | Q846W6 |
| *Streptomyces fradiae* | tylacton | TylG1 | O33954 |
|  |  | TylG2 | O33955 |
|  |  | TylG3 | O33956 |
|  |  | TylG4 | O33957 |
|  |  | TylG5 | O33958 |
| *Streptomyces hygroscopicus*  subsp*. ascomyceticus* | FK520 | FkbA | P95814 |
|  |  | FkbB | Q9ZGA4 |
|  |  | FkbC | Q9KIE1 |
| *Streptomyces hygroscopicus* | rapamycin | RapA | Q54297 |
|  |  | RapB | Q54296 |
|  |  | RapC | Q54299 |
| *Streptomyces nanchangensis* | nanchangmycin | NanA1 | Q7WTF5 |
|  |  | NanA2 | Q7WTF4 |
|  |  | NanA3 | Q7WTF3 |
|  |  | NanA4 | Q7WTF2 |
|  |  | NanA5 | Q7WTF1 |
|  |  | NanA6 | Q7WTF0 |
|  |  | NanA7 | Q7WTE3 |
|  |  | NanA8 | Q7WTD6 |
|  |  | NanA11 | Q7WTD7 |
| *Streptomyces natalensis* | pimaricin | PimS0 | Q9X992 |
|  |  | PimS1 | Q9X993 |
|  |  | PimS2 | Q9EWA1 |
|  |  | PimS3 | Q9EWA2 |
|  |  | PimS4 | Q9EWA3 |
| *Streptomyces nodosus* | amphotericin | AmphA | Q93NW8 |
|  |  | AmphB | Q93NW7 |
|  |  | AmphC | Q93NW6 |
|  |  | AmphI | Q93NX9 |
|  |  | AmphJ | Q93NX8 |
|  |  | AmphK | Q93NX7 |
| *Streptomyces noursei* | nystatin | NysA | Q9L4W5 |
|  |  | NysB | Q9L4W4 |
|  |  | NysC | Q9L4W3 |
|  |  | NysI | Q9L4X3 |
|  |  | NysJ | Q9L4X2 |
|  |  | NysK | Q9L4X1 |
| *Streptomyces* str. FR-008 | candicidin | FscA | Q9W5Q3 |
|  |  | FscB | Q6W5P9 |
|  |  | FscC | Q6W5Q0 |
|  |  | FscD | Q6W5P6 |
|  |  | FscE | Q6W5P7 |
|  |  | FscF | Q6W5P8 |
| *Streptomyces venezuelae* | pikromycin | PikAI | Q9ZGI5 |
|  |  | PikAII | Q9ZGI4 |
|  |  | PikAIII | Q9ZGI3 |
|  |  | PikAIV | Q9ZGI2 |
| *Saccharopolyspora erythrea* | erythromycin | DEBS1 | Q5UNP6 |
|  |  | DEBS2 | Q5UNP5 |
|  |  | DEBS3 | Q5UNP4 |
| *Saccharopolyspora spinosa* | spinosad | SpnA | Q9ALM6 |
|  |  | SpnB | Q9ALM5 |
|  |  | SpnC | Q9ALM4 |
|  |  | SpnD | Q9ALM3 |
|  |  | SpnE | Q9ALM2 |
| *Amycolatopsis mediterranei* | rifamycin | RifA | O54666 |
|  |  | RifB | O52545 |
|  |  | RifC | O52790 |
|  |  | RifD | O54591 |
|  |  | RifE | O54593 |
| *Micromonospora griseorubida* | mycinamicin | MycAI | Q83WF0 |
|  |  | MycAII | Q83WE9 |
|  |  | MycAIII | Q83WE8 |
|  |  | MycAIV | Q83WE7 |
|  |  | MycAV | Q83WE6 |
| *Micromonospora megalomicea* | megalomicin | MegAI | Q9F830 |
|  |  | MegAII | Q9F829 |
|  |  | MegAIII | Q9F828 |
